# *Denki buro*: a Japanese electric bath in public space causing inappropriate ICD shock

**DOI:** 10.1007/s10840-024-01832-9

**Published:** 2024-06-07

**Authors:** Masaki Hashimoto, Kenichiro Yamagata, Jun Yokota, Izumi Tanikawa, Katsuhito Fujiu

**Affiliations:** 1 Department of Cardiovascular Medicine, Graduate School of Medicine, The University of Tokyo, 7-3-1 Hongo, Bunkyo-Ku, Tokyo, 113-8655 Japan; 2 Department of Medical Engineering, The University of Tokyo Hospital, Tokyo, Japan

An 80-year-old man with a history of anteroseptal myocardial infarction underwent transvenous dual-chamber implantable cardioverter defibrillator (ICD) implantation for secondary prevention. The patient presented to our hospital after receiving an ICD shock for the first time. He had no predisposing symptoms, and the shock was delivered at 36.0 J while taking a *denki buro* (“electric bath”) (Fig. 1a). The patient immediately got out of the bath, and no further shocks occurred. Investigation of the ICD revealed low-level high-frequency noises (cycle length = 170 ms (352 bpm)) detected as ventricular fibrillation (VF) (Fig. 1b). The pacing threshold, sensitivity, and lead impedance were all within normal range. We concluded that the electric current induced by the *denki buro* caused an inappropriate ICD shock. Further investigation of the intracardiac electrogram revealed that the noise was higher in the ventricular lead than in the atrial lead, perhaps because the tip of the ventricular lead was parallel to the electrical current generated by the *denki buro* (Fig. 1a). Both noises detected in the atrial and ventricular leads fluctuated, indicating that the *denki buro* produced a current that flowed in both directions (left and right) in the patient. In addition, the noises detected simultaneously in the atrial and ventricular leads exhibited an inverted pattern in the vertical direction. This could be attributed to the reverse positioning of the positive and negative electrodes at the tip of both leads implanted in the heart (Fig. 1a). As mentioned, the noise diminished immediately upon getting out of the bath; therefore, the patient was instructed not to take *denki buro*, and no inappropriate ICD shocks have occurred since then.

**Fig. 1:**
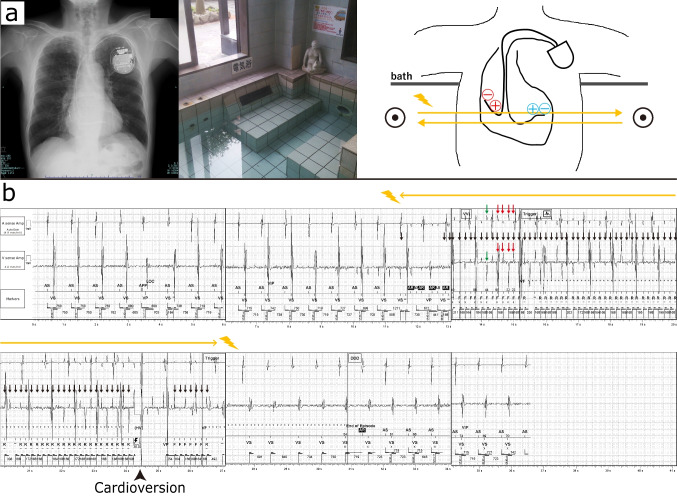
**a** A typical image of a *denki buro* and patient’s chest radiograph depicting the relative positioning of the positive and negative electrodes in the atrial and ventricular leads. Plus and minus symbols denote the positive and negative electrodes in the atrial (red) and ventricular (blue) leads. The orange arrows indicate the estimated direction of the electric current flow. **b** Implantable cardioverter defibrillator tracing of the patient. Investigation showing low-level high-frequency noises with a cycle length of 170 ms (352 bpm) (black arrows) detected as ventricular fibrillation. The noise was larger in the ventricular lead than in the atrial lead, and the simultaneously observed noises were inverted in the vertical direction (green arrows). Both noises detected in the atrial and ventricular leads fluctuated (red arrows). AS, atrial sensing; VS, ventricular sensing; AP, atrial pacing; VP, ventricular pacing; AR, atrial refractory; VF, ventricular fibrillation; F, fibrillation; R, re-detected fibrillation

Inappropriate shocks from transvenous ICD occur at a frequency of 7.3–11.5% [1, 2]. The most common cause of inappropriate shock is atrial fibrillation, while abnormal sensing accounts for 10–20% of inappropriate shocks [1]. *Denki buro* is a small bath equipped with a power supply and steel electrode plates installed on both sides of the bathtub, which discharge pulsing currents of electricity into the submerged bather. The present *denki buro* emits electricity in the form of alternating current. There is no restriction on the output frequency of *denki buro*, while the output voltage is restricted to a maximum of 10 V. The output frequency is generally set to 6–60 Hertz, depending on the company’s setting. A higher frequency current is recognized as a higher heart rate, which is at high risk of being misinterpreted as VF; however, when exceeding a certain frequency, the current may be regarded as noises due to the noise recognition function of ICD. As 6 Hertz is nearly 170 ms, ICD detected the current as VF in the current case.

*Denki buro* is widely found in public baths in Japan. The baths look similar to typical public baths, placed without an informative/cautionary sign, rendering the *denki buro* indistinguishable, at first glance, from normal baths. *Denki buro* is said to improve blood circulation by directly stimulating the muscles; however, there is no evidence to confirm this. In addition, there is a serious risk of the electric current being mistakenly regarded as VF by ICD, leading to potential inappropriate shocks. In the current case, the cycle length exceeded the detection zone for VF, causing an inappropriate ICD shock. To the best of our knowledge, this is the first reported case of inappropriate ICD shock induced by *denki buro*. Physicians should be aware of and inform patients that *denki buro* causes inappropriate shocks in individuals with ICD.

## Data Availability

The data underlying this article will be shared on reasonable request to the corresponding author.

## References

[1] Daubert JP, Zareba W, Cannom DS, et al. Inappropriate implantable cardioverter-defibrillator shocks in MADIT II: frequency, mechanisms, predictors, and survival impact. J Am Coll Cardiol. 2008;51:1357–65. 10.1016/j.jacc.2007.09.073. 18387436 10.1016/j.jacc.2007.09.073

[2] Knops RE, Olde Nordkamp LRA, Delnoy PHM, et al. Subcutaneous or transvenous defibrillator therapy. N Engl J Med. 2020;383:526–36. 10.1056/NEJMoa1915932. 32757521 10.1056/NEJMoa1915932

